# *Naegleria fowleri* in Well Water

**DOI:** 10.3201/eid1409.071076

**Published:** 2008-09

**Authors:** Barbara Blair, Payal Sarkar, Kelly R. Bright, Francine Marciano-Cabral, Charles P. Gerba

**Affiliations:** University of Arizona, Tucson, Arizona, USA (B. Blair, P. Sarkar, K.R. Bright, C.P. Gerba); Virginia Commonwealth University, Richmond, Virginia, USA (F. Marciano-Cabral)

**Keywords:** Naegleria fowleri, ameba, meningoencephalitis, protozoal, environmental exposure, letter

**To the Editor:** *Naegleria fowleri,* a protozoon found in hot springs and warm surface water, can cause primary amebic meningoencephalitis in humans. A survey of drinking water supply wells in Arizona determined that wells can be colonized and may be an unrecognized source of this organism that could present a human health risk.

*N. fowleri* is a free-living ameboflagellate found in warm bodies of water such as ponds, irrigation ditches, lakes, coastal waters, and hot springs and can cause primary amebic meningoencephalitis. Humans come into contact with *N. fowleri* by swimming or bathing, particularly in surface waters. The ameba enters the nasal passages, penetrates the nasopharyngeal mucosa, and migrates to the olfactory nerves, eventually invading the brain through the cribriform plate (*1*). From 1995 to 2004, *N. fowleri* killed 23 persons in the United States (*2*), including 2 children in the Phoenix, Arizona, area in 2002, who had been exposed to well water but had not consumed it (*3*). There have been 6 documented deaths in 2007, all in warmer regions (Arizona, Texas, Florida) (*4*).

Although *N. fowleri*’s presence in surface waters is well documented (*5*,*6*), no previous studies on its occurrence in wells have been conducted. We studied high-volume drinking water wells operated by municipal utilities or private water companies in the greater Phoenix and Tucson, Arizona, areas. Previous data from 500 wells in the region showed temperatures ranging from 13°C to 46°C. Typical well discharges ranged from hundreds to >3,780 L per minute. Well depths varied from 100 m to >300 m.

Well water samples were collected by using 1-L sterile polyethylene bottles at or near the wellhead before disinfection by well owners or utilities (*7*). In phase 1, samples were collected after wells were flushed until the water was clear. During phase 2, samples were collected as water was turned on from spigots at or near wellheads (initial) and after a 3-borehole volume had flushed through the system (purged). Additional wells were sampled during this phase. Samples were tested for temperature, pH, turbidity, chlorine residual, conductance, coliforms, heterotrophic bacterial plate counts (HPC), and *Escherichia coli* following standard methods (*7*).

To test for viable amebas, we spread aliquots on nonnutrient agar seeded with *E. coli* at 37°C (*3*,*8*). We placed scrapings from the advancing front of subsequent ameba plaques in distilled water to identify enflagellation (*5*); however, precise species identification was not possible. Live amebae were therefore harvested for PCR analysis to specifically identify *N. fowleri*. We chose PCR over the mouse pathogenicity test because other *Naegleria* species that are nonpathogenic in humans are lethal in mice (*8*). The genotype of isolates was not determined because all of the described genotypes found in the United States have been shown to be pathogenic in humans (*9*).

To concentrate trophozoites/cysts, we gently agitated samples for 2 minutes and then centrifuged and filtered them through polyethylene filters (2-μm pore; Millipore, Bedford, MA, USA). A 10-μL volume of concentrate was used as a template for nested PCR (*3*,*8*) (triplicate tests were conducted immediately and after a 2-week 37°C incubation). Positive and negative PCR products were frozen at –80°C, coded to prevent bias, and shipped to Francine Marciano-Cabral at Virginia Commonwealth University for confirmation by cloning and sequencing (*3*).

The general microbial quality of the wells was as follows: 73 (51%) had >500 HPC/mL; 8 (5.5%) were positive for coliforms; none were positive for *E. coli*. Oils used to lubricate well motors may result in the growth of HPC in well water (*10*). *N. fowleri* feeds on heterotrophic bacteria in water and could multiply in the well casing. This may explain *N. fowleri*’s colonization of wells.

The recent association in Arizona between unchlorinated drinking water and the transmission of *N. fowleri* suggests that groundwater has been an unrecognized source of this organism. PCR detected *N. fowleri* DNA in 11 (7.7%) of 143 wells. Of 185 total samples, 30 (16.2%) tested positive for *N. fowleri* (Table). The organism was most often detected after the wells had been purged (17.9% purged vs. 10.0% initial samples), suggesting that *N. fowleri* was present in the aquifer or was released from the well casing or column during pumping. The wells testing positive for *N. fowleri* ranged in temperature from 21.9°C to 37.4°C (average 29.0°C; median 29.5°C).

**Table:** *Naegleria fowleri* in well water samples, Arizona

Sample type*	No. (%) positive
Initial	4/40 (10)
Purged	26/145 (17.9)
All	30/185 (16.2)

*Samples were collected before and after purging 3 borehole volumes. PCR was used to test samples.

The live trophozoite form was confirmed in only 1 well, though 11 of 143 wells tested positive according to PCR. This discrepancy may be due to the low occurrence of trophozoites in water or to differences in assay volumes for detection of live trophozoites (0.75 mL) versus PCR (30 mL equivalent unconcentrated volume). PCR is also more sensitive, capable of detecting 100 organisms/L in an unconcentrated sample (*8*); however, PCR did not determine if the amebas were infectious. Although PCR can determine the species by using primers for a specific gene sequence not found in other *Naegleria* species, it cannot determine the life stage (cyst/trophozoite). Trophozoites are believed to be the infectious form of the organism (*1*); nonetheless, cysts can be equally harmful because they may revert to trophozoites under optimal conditions (*1*). The surprisingly common occurrence of *N. fowleri* in drinking water wells suggests that groundwaters may be an unrecognized human health threat.
